# Peroxiredoxin 1 Stimulates Endothelial Cell Expression of VEGF via TLR4 Dependent Activation of HIF-1α

**DOI:** 10.1371/journal.pone.0050394

**Published:** 2012-11-21

**Authors:** Jonah R. Riddell, Patricia Maier, Stephanie N. Sass, Michael T. Moser, Barbara A. Foster, Sandra O. Gollnick

**Affiliations:** 1 Department of Cell Stress Biology, Roswell Park Cancer Institute, Buffalo, New York, United States of America; 2 Department of Immunology, Roswell Park Cancer Institute, Buffalo, New York, United States of America; 3 Department of Pharmacology and Therapeutics, Roswell Park Cancer Institute, Buffalo, New York, United States of America; University of Nebraska Medical Center, United States of America

## Abstract

Chronic inflammation leads to the formation of a pro-tumorigenic microenvironment that can promote tumor development, growth and differentiation through augmentation of tumor angiogenesis. Prostate cancer (CaP) risk and prognosis are adversely correlated with a number of inflammatory and angiogenic mediators, including Toll-like receptors (TLRs), NF-κB and vascular endothelial growth factor (VEGF). Peroxiredoxin 1 (Prx1) was recently identified as an endogenous ligand for TLR4 that is secreted from CaP cells and promotes inflammation. Inhibition of Prx1 by CaP cells resulted in reduced expression of VEGF, diminished tumor vasculature and retarded tumor growth. The mechanism by which Prx1 regulates VEGF expression in normoxic conditions was investigated in the current study. Our results show that incubation of mouse vascular endothelial cells with recombinant Prx1 caused increases in VEGF expression that was dependent upon TLR4 and required hypoxia inducible factor-1 (HIF-1) interaction with the VEGF promoter. The induction of VEGF was also dependent upon NF-κB; however, NF-κB interaction with the VEGF promoter was not required for Prx1 induction of VEGF suggesting that NF-κB was acting indirectly to induce VEGF expression. The results presented here show that Prx1 stimulation increased NF-κB interaction with the HIF-1α promoter, leading to enhanced promoter activity and increases in HIF-1α mRNA levels, as well as augmented HIF-1 activity that resulted in VEGF expression. Prx1 induced HIF-1 also promoted NF-κB activity, suggesting the presence of a positive feedback loop that has the potential to perpetuate Prx1 induction of angiogenesis. Strikingly, inhibition of Prx1 expression in CaP was accompanied with reduced expression of HIF-1α. The combined findings of the current study and our previous study suggest that Prx1 interaction with TLR4 promotes CaP growth potentially through chronic activation of tumor angiogenesis.

## Introduction

Numerous studies have shown that inflammatory conditions increase the risk of cancer [1]–[3]. In established tumors inflammation is a key component of the tumor microenvironment that contributes to tumor survival, proliferation and invasion [4]. Hallmarks of inflammation in cancer include persistent NF-κB activation and increased angiogenesis as a result of elevated levels of vascular endothelial growth factor (VEGF). Prostate tumors express constitutively activated NF-κB [5], [6] and elevated VEGF levels [7] that are associated with disease progression [8]–[12]. The factors that promote chronic inflammation in established prostate cancer (CaP) are largely unknown. We have recently identified peroxiredoxin 1 (Prx1) as a mediator of inflammation in CaP [13].

Prx1 is an intracellular peroxidase and chaperone protein that is non-classically secreted from prostate tumor cells [14]–[16]. Extracellular Prx1 is a Toll-like receptor 4 (TLR4) ligand [15]. Binding of Prx1 to TLR4 stimulates release of pro-inflammatory cytokines interleukin (IL)-6 and tumor necrosis factor (TNF)-α from macrophages [15] and VEGF from tumor cells, macrophages and endothelial cells [13]. Inhibition of Prx1 expression in prostate tumor cells resulted in decreased subcutaneous tumor growth and low levels of VEGF that were associated with reduced functional tumor vasculature and vessel number [13]. The mechanism of by which Prx1 regulates VEGF is undefined.

VEGF expression is elevated in hypoxic conditions. Elevated VEGF expression is a result of increased transcriptional activity that is mediated by the transcription factor HIF-1 (hypoxia inducible factor-1) [17]. HIF-1 is comprised of two subunits, a constitutively expressed β subunit and an inducible labile α subunit. In non-inflammatory, normoxic conditions HIF-1α is associated with prolyl hydroxylases (PHD-1, -2, -3) and von Hippel-Lindau (pVHL)-E3 ligase complexes that cause rapid proteosome mediated degradation of HIF-1α. In settings of reduced oxygen PHD activity is reduced allowing for translocation of HIF-1α to the nucleus where it interacts with HIF-1β and binds to hypoxia-response elements (HRE) to simulate target gene transcription [18]. Several studies have shown that pro-inflammatory cytokines and TLR signaling can induce HIF-1α gene expression and increase HIF-1α stability in normoxic conditions [18]. Lipopolysaccharide (LPS), the prototypical exogenous TLR4 ligand, can induce VEGF and HIF-1α gene expression via activation of NF-κB in macrophages [19]–[22], and dendritic cells [23], [24]. Other studies have demonstrated that generation of reactive oxygen rather than NF-κB is responsible for TLR4 mediated increases in VEGF and HIF-1α mRNA and protein levels in macrophages [25]. Finally some studies indicate that the LPS:TLR4 interaction alone is insufficient for induction of VEGF and HIF-1α and requires simultaneous activation of the adenosine A_2A_ receptor [22], [26]. Thus the mechanism of induction of VEGF by TLR4 agonists is somewhat controversial and warrants additional study.

Activation of VEGF expression by TLR4 may be cell type specific. For example, LPS:TLR4 interaction induces VEGF expression in fibroblasts without increasing expression of HIF-1α [27], while induction in macrophages is dependent upon HIF-1α. The tumor microenvironment consists of tumor cells, extracellular matrix and host cell populations, including endothelial cells and their precursors, pericytes, muscle cells, fibroblasts and inflammatory cells [1], [28]. Previous study suggested that Prx1 stimulation of VEGF expression from host cells was important for angiogenesis [13].

In this study we examined the mechanism by which Prx1 stimulates VEGF expression and migration in endothelial cells. We demonstrate that Prx1 regulates endothelial cell VEGF protein expression, mRNA levels, and promoter activity in a TLR4 signaling dependent manner. Analysis of VEGF promoter activity revealed that Prx1 induction of VEGF was dependent upon HIF-1 interaction with the hypoxic response element (HRE) within the VEGF promoter. Subsequent studies showed that Prx1 and TLR4 interaction increases HIF-1α mRNA expression, promoter activity and protein levels in a NF-κB dependent manner. Additionally, Prx1 and TLR4 interaction leads to a MEK dependent eIF-4E phosphorylation, which may mediate increases in transcription and/or translation of HIF-1α. Interestingly, tumors with reduced Prx1 levels express lower levels of HIF-1α. Prx1 stimulation of VEGF expression results in endothelial cell proliferation and motility that is dependent upon TLR4, NF-κB, and HIF-1.

## Results

### Prx1 Stimulates Endothelial Cell Expression of VEGF

**Figure 1:**
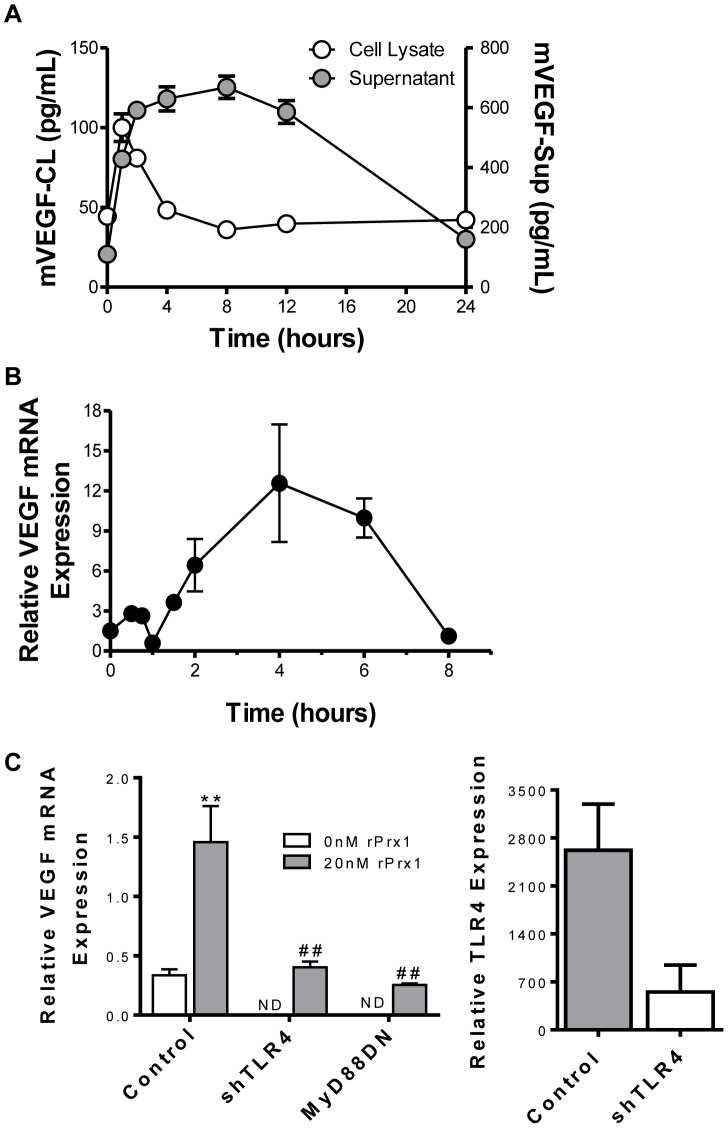
Prx1 stimulates endothelial cell expression of VEGF. 2H-11 cells were stimulated with 20 nM rPrx1 for various times. **A.** Culture media and cell lysates were harvested at 1, 2, 4, 8, 12 and 24 hours; VEGF protein levels were determined by ELISA. The mean values ± SD of 3 experiments are shown. The zero time point corresponds to VEGF levels present in untreated control cells. **B.** Total RNA was collected at 0.5, 1, 2, 4 and 6 hours; VEGF mRNA levels were determined by performing qPCR. A representative experiment of 3 repeats is shown. Results are presented as the average relative VEGR mRNA expression. The zero time point corresponds to VEGF levels present in untreated control cells. **C.** 2H-11 cells were transfected with control vectors (Control) or with vectors engineered to express shTLR4 or MyD88DN. The effect of shRNA on TLR4 expression is shown on the right. Cells were treated with rPrx118 hours after transfection; RNA was collected after 6 hours of stimulation and VEGF mRNA levels were measured by qPCR. Results are presented as the average relative VEGR mRNA expression. Error bars are ± SEM; n≥3. ** Represents P≤0.01 when compared to control untreated levels; ## represents P≤0.01 when compared to control stimulated values. ND = not done.

The effect of rPrx1 on endothelial cell expression of VEGF protein and mRNA expression was tested *in vitro*. rPrx1 stimulated maximal VEGF expression in 2H-11 murine endothelial cell lysates within an hour (Figure 1A). VEGF expression declined to baseline 4 hours after stimulation. VEGF secretion increased within 2 hours of stimulation. Levels remained high for 12 hours and declined to baseline by 24 hours (Figure 1A). rPrx1 induced an early increase in VEGF mRNA levels that peaked within 45 minutes and rapidly declined by 1 hour (Figure 1B). A second more prominent increase was observed 2 h after simulation and was maximal at 4 h. To determine whether the induction of VEGF in endothelial cells by rPrx1 was dependent upon TLR4 expression or signaling endothelial cells were transfected with plasmids encoding either a shRNA specific for TLR4 (shTLR4) or a dominant negative mutant of MyD88 (MyD88DN), that abrogates TLR4 signaling [29]. Transfection with shTLR4 resulted in a greater than 80% reduction in TLR4 expression as determined by flow cytometry; MyD88DN expression caused loss of TLR4 signaling (Figure 1C and [13]). Endothelial cell expression of shTLR4 or MyD88DN prior to rPrx1 stimulation abolished the increase in VEGF mRNA levels (Figure 1C); therefore, Prx1 induction of VEGF is dependent upon both TLR4 and TLR signaling.

### Prx1 Induction of VEGF is Dependent on HIF-1

**Figure 2:**
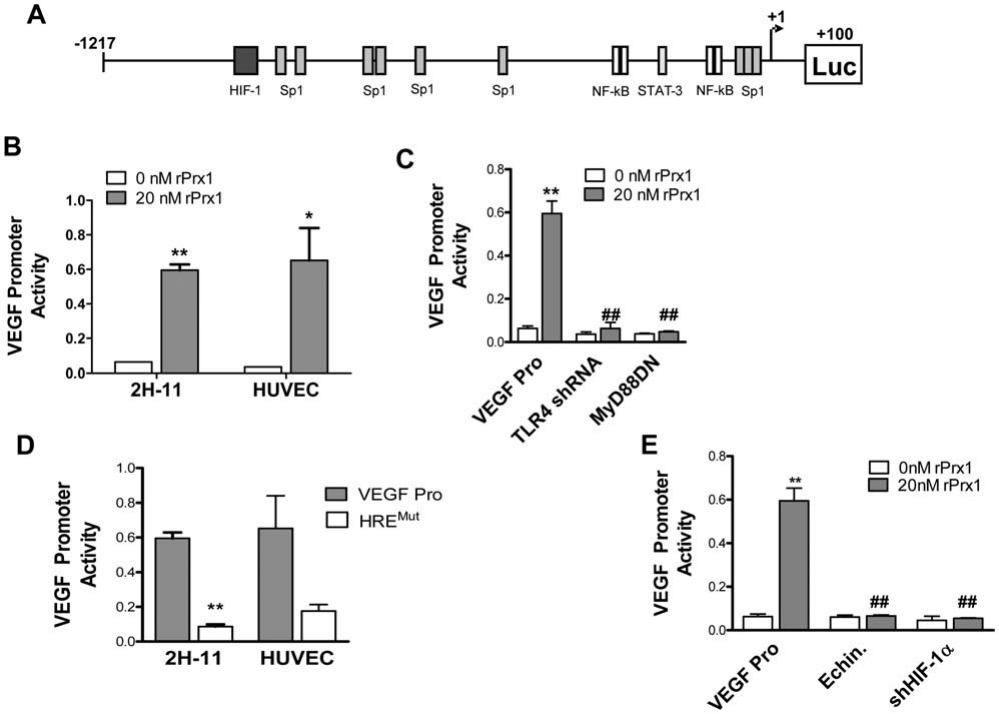
Prx1 induction of VEGF is dependent upon HIF-1. **A.** The mouse VEGF promoter is depicted with selected promoter elements including sites for HIF-1, Sp1, NF-κB and STAT-3. **B.** 2H-11 and HUVEC cells were transfected with the VEGF promoter reporter plasmid (VEGF Pro) in the absence or presence of vectors engineered to express shTLR4 or a MyD88DN. Cells were stimulated with 20 nM Prx1 24 hours after transfection and VEGF promoter activity was measured 6 hours later. Results are presented as the average relative VEGF promoter activity. Error bars represent SEM; n≥3. **B.** **2H**-11 cells were transfected with the VEGF promoter reporter plasmid (VEGF Pro) in the absence or presence of vectors engineered to express shTLR4 or a MyD88DN. Cells were stimulated with 20 nM Prx1 24 hours after transfection and VEGF promoter activity was measured 6 hours later. Results are presented as the average relative VEGF promoter activity. Error bars represent SEM; n≥3. **D.** 2H-11 cells were transfected with the VEGF promoter reporter plasmid (VEGF Pro) or a VEGF promoter reporter plasmid in which the HRE was mutated (HRE^MUT^). Cells were stimulated with 20 nM Prx1 24 hours after transfection and VEGF promoter activity was measured 6 hours later. Results are presented as the average relative VEGF promoter activity; error bars represent SEM; n≥3. ND = not done. **E.** 2H-11 cells were transfected with the VEGF Pro in the absence or presence of a vector engineered to express shHIF-1α. Cells were stimulated with 20 nM Prx1 24 hours after transfection and VEGF promoter activity was measured 6 hours later. In some cases cells were treated with echinomycin (Echin) for 16 h prior to addition of rPrx1. Results are presented as the average relative VEGF promoter activity. Error bars represent SEM; n≥3. ** Represents P≤0.01 when compared to control untreated levels; ## represents P≤0.01 when compared to control stimulated values.

The VEGF promoter contains elements for specific protein 1 (Sp1), HIF-1, NF-κB and STAT-3 (Figure 2A) [30]. HIF-1 interaction with the HRE in the VEGF promoter has been implicated in LPS induction of VEGF in macrophages and dendritic cells [20], [21]. The effect of rPrx1 on VEGF promoter activity was examined in 2H-11 endothelial cells using a luciferase reporter assay. The VEGF promoter region (−1217 bp to +100 bp) was inserted upstream of the luciferase gene (VEGF Pro). 2H-11 cells were transiently transfected with the VEGF/Luciferase promoter construct alone or in combination with plasmids encoding shTLR4 or MyD88DN. Incubation with rPrx1 increased VEGF promoter activity in 2H-11 and HUVEC cells (Figure 2B). Expression of shTLR4 or MyD88DN prior to Prx1 stimulation of 2H-11 cells abolished the enhanced VEGF promoter activity (Figure 2C). To determine if Prx1 control of the VEGF promoter was mediated by HRE activation, the HRE element in the VEGF promoter was mutated (HRE^MUT^). Mutation of the HIF-1 binding site abrogated stimulation of VEGF promoter activity by Prx1 in both 2H-11 and HUVEC (Figure 2D). To verify that HIF-1 was driving VEGF promoter activity following Prx1 stimulation, 2H-11 cells were treated with echinomycin, an inhibitor of HIF-1 interaction with the HRE, or cells were transfected with a vector engineered to express shRNA specific to HIF-1α expression (shHIF-1α) prior to stimulation with Prx1; expression of shHIF-1α led to a 70–80% reduction in HIF-1α levels. Inhibition of HIF-1 activity or shHIF-1α expression resulted in abolishment of Prx1 induced VEGF promoter activity (Figure 2E).

**Figure 3:**
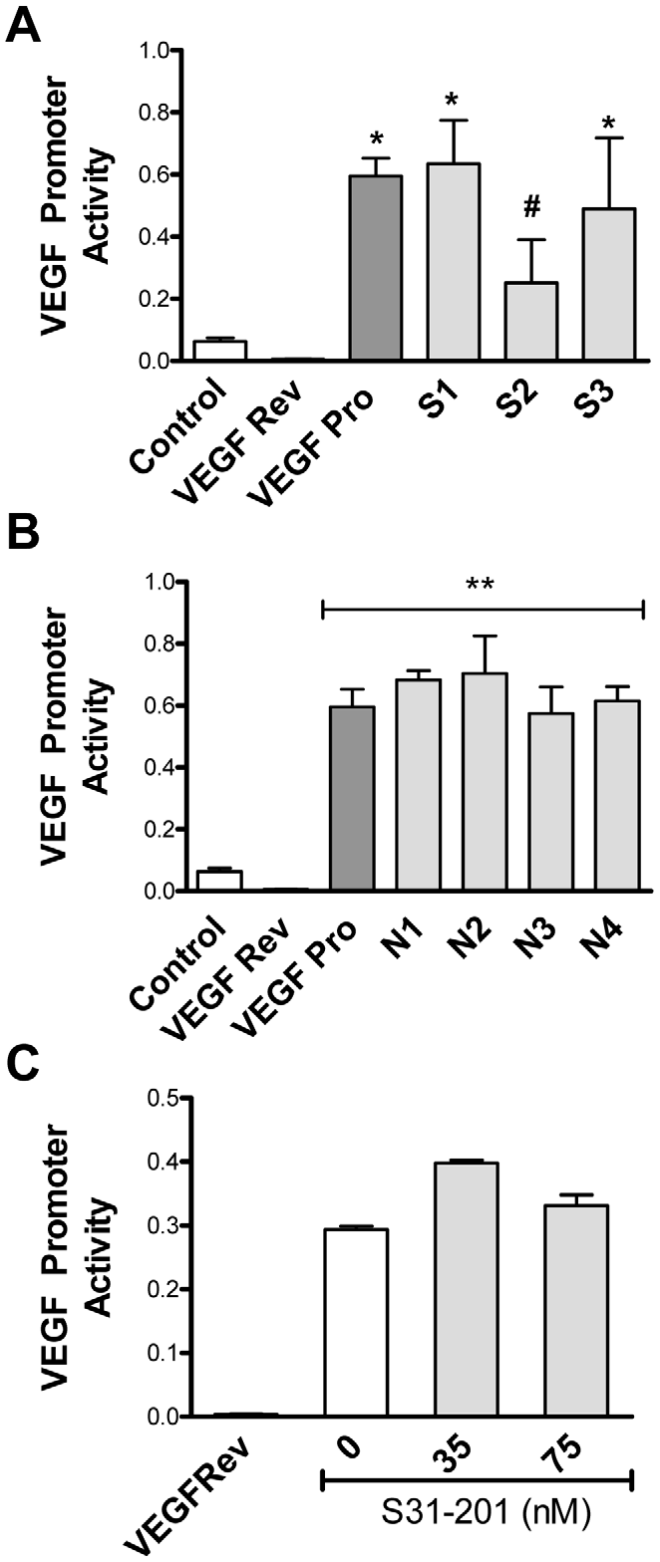
Direct stimulation of VEGF promoter activity by Prx1 depends on Sp1 but not NF-κB or STAT-3. 2H-11 cells were transfected with the VEGF promoter luciferase reporter construct (VEGF Pro), a control in which the VEGF promoter was reversed (VEGF Rev) or with VEGF promoter constructs mutated at either the (**A**) SP1 sites (S1: −75 to −61; S2: −65 to −51; S3 −52 to −38) or (**B**) NF-κB sites (N1/N2: −227 to −213; N3/4: −120 to −106). Cells were either untreated (Control) or stimulated with 20 nM Prx1 24 hours after transfection and promoter activity was measured 6 hours later. Results are presented as the average relative VEGF promoter activity; error bars represent SEM. **C.** 2H-11 cells were transfected with a control in which the VEGF promoter was reversed (VEGF Rev) or with the VEGF promoter luciferase reporter construct (VEGF Pro). Transfected cells were treated with S31–201 for 3 hours followed by treatment with 20 nM rPrx1 for 6 hours. Promoter activity was measured. Results are presented as the average relative VEGF promoter activity; error bars represent SEM. * Represents P≤0.05 when compared to untreated control levels. ** Represents P≤0.01 when compared to untreated control levels; # represents P≤0.05 when compared to VEGF Pro stimulated values.

The three Sp1 elements in the 0–85 bp region of the VEGF promoter (S1: −75 to −61; S2: −65 to −51; S3: −52 to −38 bps) are necessary for basal activity [31], [32]. HIF-1 can act in conjunction with these elements to stimulate VEGF promoter activity [33]. To test the involvement of Sp1 in Prx1 regulation of the VEGF promoter, each of the Sp1 sites was mutated and the effect of the mutation on VEGF promoter activity following Prx1 stimulation was measured. Mutation of the S2 site (−65 to −51 bp) reduced Prx1 activation of the VEGF promoter (Figure 3A). Sp1 involvement in Prx1 regulation of VEGF promoter activity was confirmed using Mithramycin A, a Sp1 inhibitor. Mithramycin A treatment of 2H-11 cells abrogated Prx1 stimulation of VEGF promoter activity (data not shown).

Activation of the TLR4 signaling cascade in macrophages by Prx1 culminates in the activation of NF-κB [15]. The VEGF promoter contains four punitive NF-κB consensus binding sites, N1/2**:** −227 to −213 and N3/4: −120 to −106 (Figure 2A). It is possible that Prx1 stimulation of VEGF promoter activity is due in part to NF-κB. To assess whether NF-κB was regulating Prx1 induction of VEGF the NF-κB regulatory elements were mutated. Mutation of the NF-κB sites had no effect on rPrx1 stimulation of VEGF promoter activity in endothelial cells (Figure 3B).

HIF-1 can also act in conjunction with STAT3 to control VEGF promoter activity [34]. To investigate the role of STAT3 in Prx1 activation of the VEGF promoter 2H-11 cells were transiently transfected with the VEGF promoter reporter constructs, treated with S31–201 (NSC 74859), and then rPrx1. S31–201 is a chemical inhibitor of STAT3 activity [35]. Incubation with S31–201 had no effect on rPrx1 induction of VEGF promoter activity (Figure 3C). Thus, Prx1 regulates VEGF promoter activity through HIF-1 interaction with the HRE and independently of the NF-κB and STAT-3 regulatory elements.

### Prx1 Enhances HIF-1 Activity

**Figure 4:**
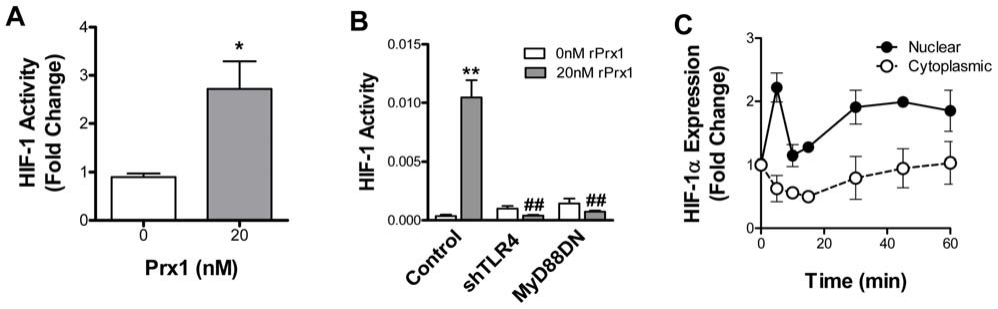
Prx1 regulates HIF-1 activity. **A.** 2H-11 endothelial cells were stimulated with rPrx1; cells were harvested at 1 hour. ChIP assays were performed by immunoprecipitation with antibodies to HIF-1α and qPCR amplification of isolated DNA fragments with VEGF promoter specific primers. Results are normalized to qPCR levels of the VEGF promoter prior to enrichment through IP. Results are reported as a fold change to untreated cells. Error bars represent SEM; n≥3. **B.** 2H-11 cells were transfected with a HIF-1 responsive firefly luciferase promoter construct containing three HRE sites (Control) in the presence or absence of plasmids encoding shTLR4 or MyD88. Transfected cells were treated with rPrx1 (20 nM); luciferase assays were performed at 6 h. Results are presented as the average relative HIF-1 activity; error bars represent SEM; n≥3. * represents P≤0.05 when compared to control untreated levels;** represents P≤0.01 when compared to control untreated levels; ## represents P≤0.01 when compared to control stimulated values. **C.** 2H-11 cells were treated with 20 nM rPrx1; nuclear and cytoplasmic fractions were isolated from cells at the indicated time points. Cell fractions were separated by electrophoresis and probed for expression of HIF-1α, laminin β1 and α-Tubulin. Fold increase in HIF-1α protein expression relative to the untreated control is shown. Error bars represent SEM.

The studies described in the previous section suggest that Prx1 enhances HIF-1 activity. To confirm this hypothesis, chromatin immunoprecipitation (ChIP) assays of the VEGF promoter using antibodies specific to HIF-1α and qPCR primers specific to the VEGF promoter region containing the HRE site were performed. rPrx1 stimulation of 2H-11 cells led to increase HIF-1 interaction with the VEGF promoter (Figure 4A). HIF-1 activity following Prx1 stimulation was further assessed using a HIF-1 dependent promoter reporter containing three HRE elements inserted upstream of luciferase. The HIF-1 reporter was expressed in 2H-11 cells in the presence or absence of plasmids encoding either shTLR4 or MyD88DN. Stimulation with rPrx1 resulted in increased promoter activation. Expression of shTLR4 or MyD88DN abolished the Prx1 induced HIF-1 activity (Figure 4B). Stimulation of 2H-11 cells with rPrx1 also increased translocation of HIF-1 to the nucleus (Figure 4C). These results indicate that Prx1 increases HIF-1 activity and interaction with the VEGF promoter in a TLR4, MyD88 dependent manner.

### Prx1 Regulates HIF-1α Expression

**Figure 5:**
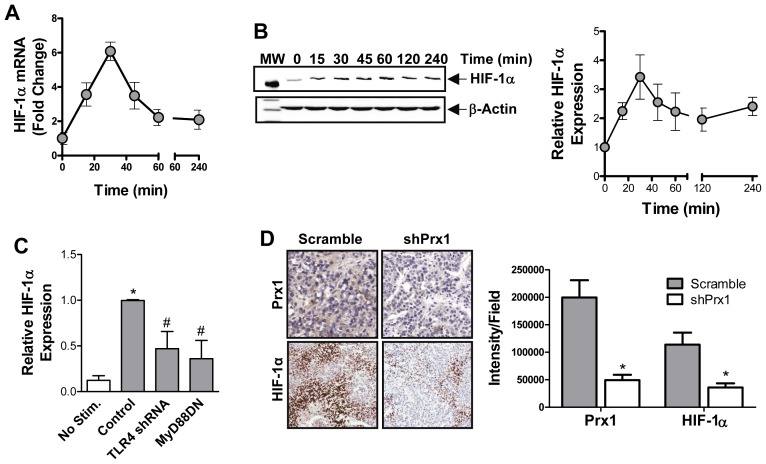
Prx1 regulates HIF-1α expression. **A.** 2H-11 cells were stimulated with 20 nM rPrx1. Total RNA was collected at various times after stimulation and qPCR was performed with HIF-1α specific primers. The mean fold change in HIF-1α mRNA expression relative to levels present in untreated cells is shown. Error bars represent SEM; n≥3. **B.** Endothelial cells were stimulated with rPrx1 (20 nM) for the indicated time periods; cell lysates were generated and a Western blot was performed with antibodies specific to HIF-1α and β-Actin. Quantization of HIF-1α expression from 3 experiments is shown (right); error bars represent SEM. **C.** 2H-11 cells were transfected with control vector or vectors engineered to express shTLR4 and MyD88DN. Cell lysates were generated from untreated (0 nM rPrx1) and treated (20 nM rPrx1) transfected cells 45 minutes after stimulation. HIF-1α levels were determined by Western analysis. Results are depicted as the mean HIF-1α levels from three experiments; error bars represent SEM. **D.** PC-3M scramble and shPrx1 tumors were harvested from mice when they reached 150 mm^3^. Prx1 and HIF-1α expression was determined by IHC. Quantization of the IHC is shown on the right and represented as HIF-1α expression (Intensity) per field. * Represents P≤0.05 when compared to control untreated levels; # represents P≤0.05 when compared to control stimulated values or levels in scramble PC-3M tumors.

TLR4 signaling can lead to increased HIF-1α expression in macrophages [19], [23], [24], but was shown to have no effect on fibroblast expression of HIF-1α mRNA [27]. To determine if Prx1 induces HIF-1-α in endothelial cells, qPCR using HIF-1α specific primers was performed on rPrx1 stimulated 2H-11 cells. HIF-1α mRNA levels in 2H-11 cells peaked 20 min after stimulation with rPrx1 (Figure 5A). Stimulation of 2H-11 endothelial cells with rPrx1 induced an increase in HIF-1α protein expression that reaches maximal by 30 minutes (Figure 5B). HIF-1α levels remained above baseline levels for at least 2 h. Prx1 mediated increases in HIF-1α was dependent upon TLR4 expression and signaling as prior expression of shTLR4 or MyD88DN abolished the effect of Prx1 on HIF-1α (Figure 5C).

Our previous study indicated that Prx1 expression in prostate tumors correlated with increased VEGF expression [13]. To determine whether HIF-1α expression also correlated with Prx1 expression in prostate tumors, immunohistochemistry was performed. Equal size (150 mm^3^) PC-3M prostate tumors expressing non-specific shRNA (Scramble) or shRNA specific for Prx1 (shPrx1) were collected and examined for Prx1 and HIF-1α expression. Reduction of Prx1 expression in PC-3M tumors correlated with reduced the HIF-1α expression tumors (Figure 5D). Thus Prx1 enhanced expression of HIF-1α in endothelial cells *in vitro* and in prostate tumors *in vivo*.

### Prx1 Enhances HIF-1 Activity via NF-κB

**Figure 6:**
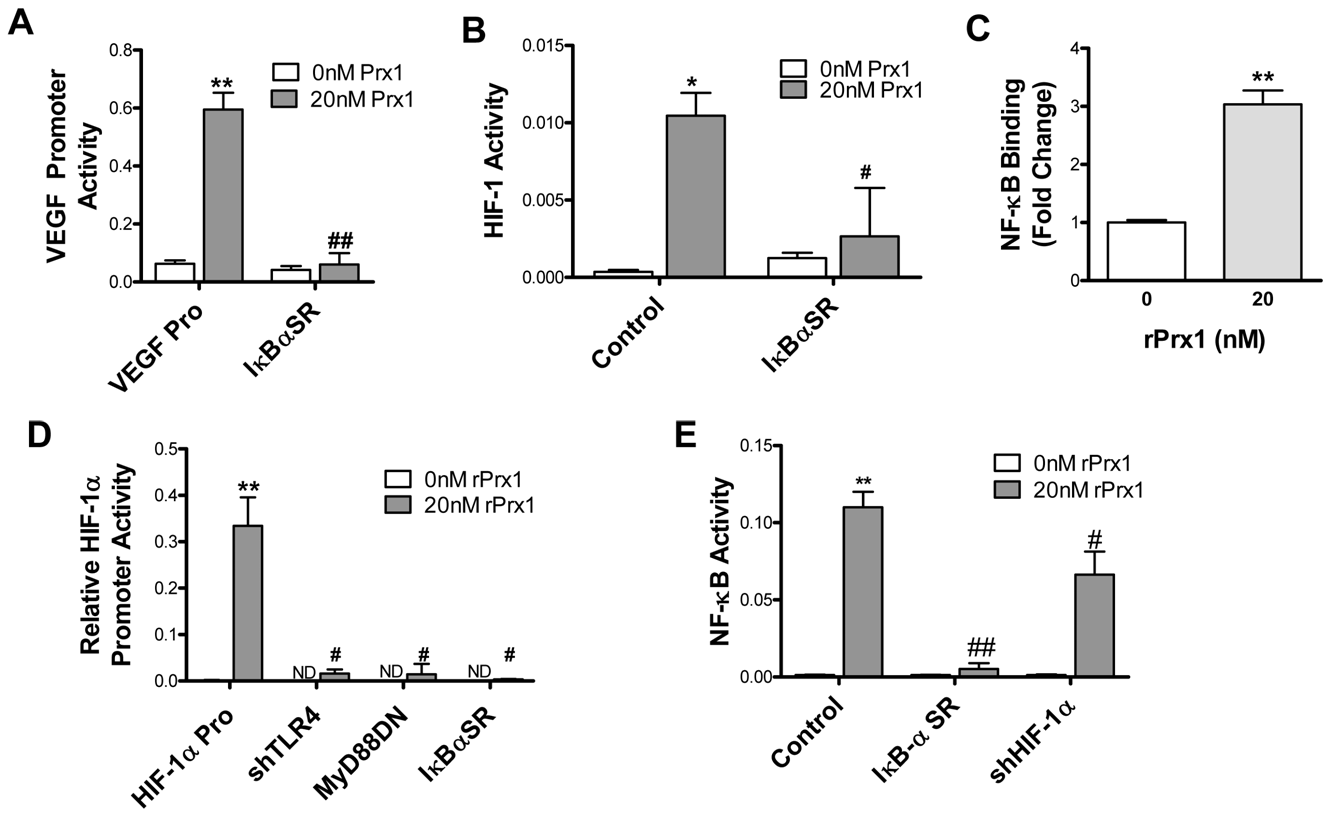
Prx1 stimulates HIF-1α expression and HIF-1 activity through NF-κB. **A.** 2H-11 cells were transfected with the VEGF promoter luciferase reporter construct alone (VEGF Pro) or with a vector encoding for IκB-αSR. Cells were stimulated with 20 nM Prx1 24 hours after transfection and VEGF promoter activity was measured 6 hours later. Results are presented as the average relative VEGF promoter activity; error bars represent SEM. **B.** HIF-1 activity was assessed using the HIF-1 responsive luciferase reporter. 2H-11 endothelial cells were transfected with the HIF-1 reporter alone (Control) or with an expression vector for an IκB-α SR. Cells were stimulated with 20 nM Prx1 24 hours after transfection and VEGF promoter activity was measured 6 hours later. Results are presented as the average relative VEGF promoter activity. Error bars represent SEM; n≥3. **C.** 2H-11 endothelial cells were stimulated with rPrx1; cells were harvested at 1 hour. ChIP assays were performed by immunoprecipitation with antibodies to NF-κB and qPCR amplification of isolated DNA fragments with HIF-1α promoter specific primers. Results are normalized to qPCR levels of the HIF-1α promoter prior to enrichment through IP. Results are reported as a fold change to untreated cells. Error bars represent SEM; n≥3. **D.** 2H-11 endothelial cells were transfected with the HIF-1α promoter (HIF-1α Pro) alone or with an expression vectors encoding for shRNA specific for TLR4 (shTLR4), MyD88DN or IκB-αSR. Cells were stimulated with 20 nM Prx1 24 hours after transfection and HIF-1α promoter activity was measured 6 hours later. Results are presented as the average relative HIF-1α promoter activity; error bars represent SEM; n≥3. ND = not done. **D.** 2H-11 cells were transfected with NF-κB responsive reporter construct alone (Control) or with vectors encoding for IκB-αSR or shHIF-1α. Cells were stimulated with 20 nM Prx1 24 hours after transfection and NF-κB responsive reporter activity was measured 6 hours later. Results are presented as the average relative NF-κB activity; error bars represent SEM. ** Represents P≤0.01 when compared to control untreated levels; # represents P≤0.05 and ## represents P0.01 when compared to Control stimulated values. N≥3.

The VEGF promoter contains four putative NF-κB regulatory sites that were not directly involved in Prx1 activation of the VEGF promoter (Figure 3B). To determine if NF-κB was indirectly involved in Prx1 induced VEGF promoter activity, 2H-11 cells were transiently transfected with a plasmid encoding a mutant form of IκB-α that is a constitutively phosphorylated and therefore acts as a super repressor of NF-κB (IκB-αSR). Expression of IκB-αSR inhibited Prx1 induction of VEGF promoter activity (Figure 6A), suggesting that Prx1 activation of NF-κB indirectly regulates VEGF expression.

In some studies, but not all TLR4 stimulated increases in HIF-1α have been shown to be due to NF-κB dependent increases in transcription of HIF-1α [18]. To test if Prx1 mediated increases in HIF-1 activity and HIF-1α expression were NF-κB dependent, 2H-11 cells were transiently transfected with a HIF-1 reporter plasmid in the presence or absence of IκB-αSR. Endothelial cell expression of the IκB-αSR prior to stimulation prevented Prx1 induction of HIF-1 activity (Figure 6B), as well as increases in HIF-1α protein and mRNA expression (data not shown). To determine if Prx1 stimulation increased NF-κB interaction with the HIF-1α promoter region, a ChIP assay was performed by immunoprecipitation with antibodies specific for NF-κB p65 and qPCR with primers specific to the HIF-1α promoter. NF-κB interaction with the HIF-1α promoter was increased following stimulation with rPrx1 (Figure 6C). Prx1 stimulation of 2H-11 cells also resulted in increased HIF-1α promoter activity (Figure 6D). Expression of shTLR4, MyD88DN, or IκB-αSR abrogated the Prx1 induction of HIF-α promoter activity (Figure 6D). Results demonstrate that Prx1 and TLR4 interaction regulates HIF-1α promoter activity and HIF-1 activity in an NF-κB dependent manner.

NF-κB has been identified as a HIF-dependent target; HIF-1 can increase transcription of the p65 subunit of NF-κB and increase NF-κB activity in neutrophils [36]. HIF-1 also causes hyperphosphorylation of IκB and phosphorylation of p65, which leads to enhanced nuclear translocation and increased NF-κB activity [37]. To determine if Prx1 induction of HIF-1 augmented NF-κB activity 2H-11 cells were transfected with a NF-κB reporter plasmid in the presence or absence a plasmid encoding shRNA specific for HIF-1α (shHIF-1α). Inhibition of HIF-1α reduced Prx1 enhanced NF-κB activity suggesting the presence of a positive feedback loop between NF-κB and HIF-1α following Prx1 stimulation of endothelial cells (Figure 6E).

### Prx1 Activation of ERK and eIF-4E is Required for HIF-1α Induction

**Figure 7:**
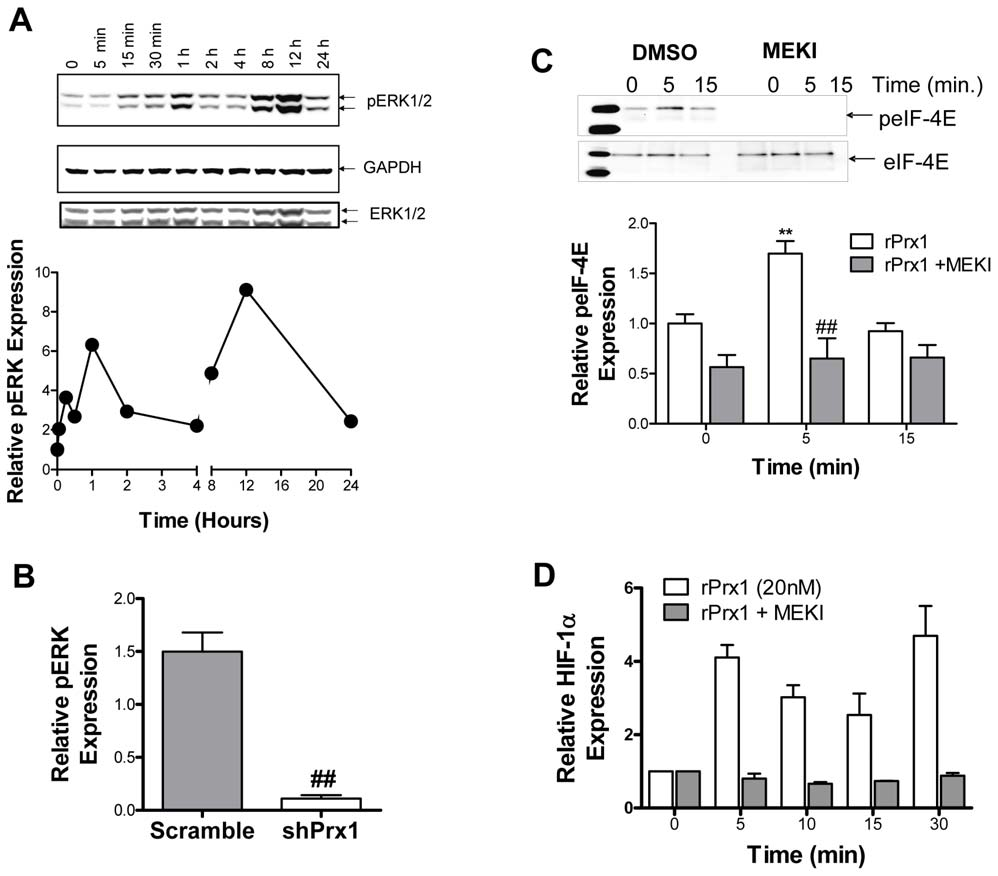
Prx1 induces phosphorylation of ERK *in vitro* and *in vivo*. **A.** 2H-11 endothelial cells were stimulated with rPrx1 (20 nM) for the indicated time periods; cell lysates were generated and a Western blot was performed with antibodies specific to pERK1/2, total ERK, and GAPDH. A representative blot is shown; quantization of relative pERK expression from 3 experiments is shown on the right. **B.** PC-3M scramble and shPrx1 tumors were harvested at 150 mm^3^. Tumor lysates were separated by electrophoresis and stained with antibodies specific to phosphorylated ERK1/2, total ERK1/2, β-Actin and Prx1. A representative blot is shown; quantization of relative pERK expression is shown on the right. Error bars represent SEM; ## represents P≤0.01.

**Figure 8:**
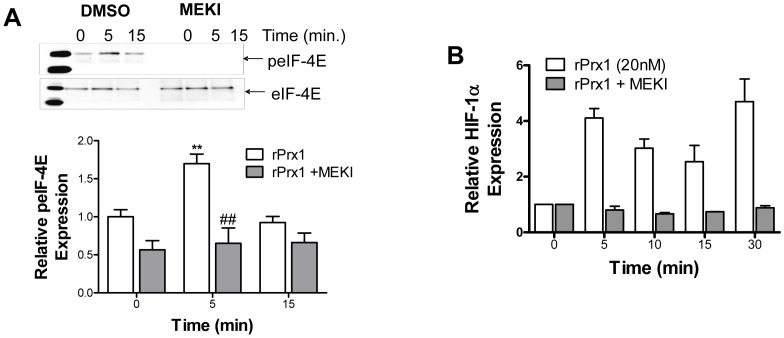
Prx1 Activation of ERK and eIF-4E is Required for HIF-1α Induction. **A/B.** 2H-11 cells were treated with DMSO or U0126 (MEKI) prior to Prx1 stimulation. **C.** Cell lysates were generated and total eIF-4E was isolated by immunoprecipitation. Fractions were separated by electrophoresis and stained for expression of phosphorylated or total eIF-4E. A representative blot is shown; quantization of relative peIF-4E expression from 3 experiments is shown below; error bars represent SEM, n = 3. **D.** Cell lysates were generated and a Western blot was performed with antibodies specific to HIF-1α and GAPDH. Quantization of relative HIF-1α expression from 3 experiments is shown; error bars represent SEM. ** Represents P≤0.01 when compared to control untreated levels; ## represents P≤0.01 when compared to control stimulated values.

ERK is phosphorylated and activated upon TLR4 activation [38]–[40]. Activated ERK can cause increased translation of HIF-1α mRNA through activation of the eukaryotic translation initiation factor 4E (eIF-4E) [41]. The effect of Prx1 on ERK activation was examined *in vitro* and *in vivo*. Prx1 stimulation of 2H-11 endothelial cells induced phosphorylation of ERK (Figure 7A). Reduction of Prx1 expression in PC-3M tumors is associated with reduced levels of phosphorylated ERK (Figure 7B). Prx1 stimulation of endothelial cells also increased phosphorylation of eIF-4E (Figure 8A). Inhibition of ERK1/2 in endothelial cells by the MEK1/2 inhibitor U2106 abolished Prx1 mediated increases in phosphorylation of eIF-4E and HIF-1α protein expression (Figure 8A/B). These data suggest that Prx1 may be regulating HIF-1α translation; however it is also possible that activated Erk directly activates HIF-1α transcription.

### Prx1 Induction of Endothelial Cell Proliferation and Migration Depends on NF-κB and HIF-1

**Figure 9:**
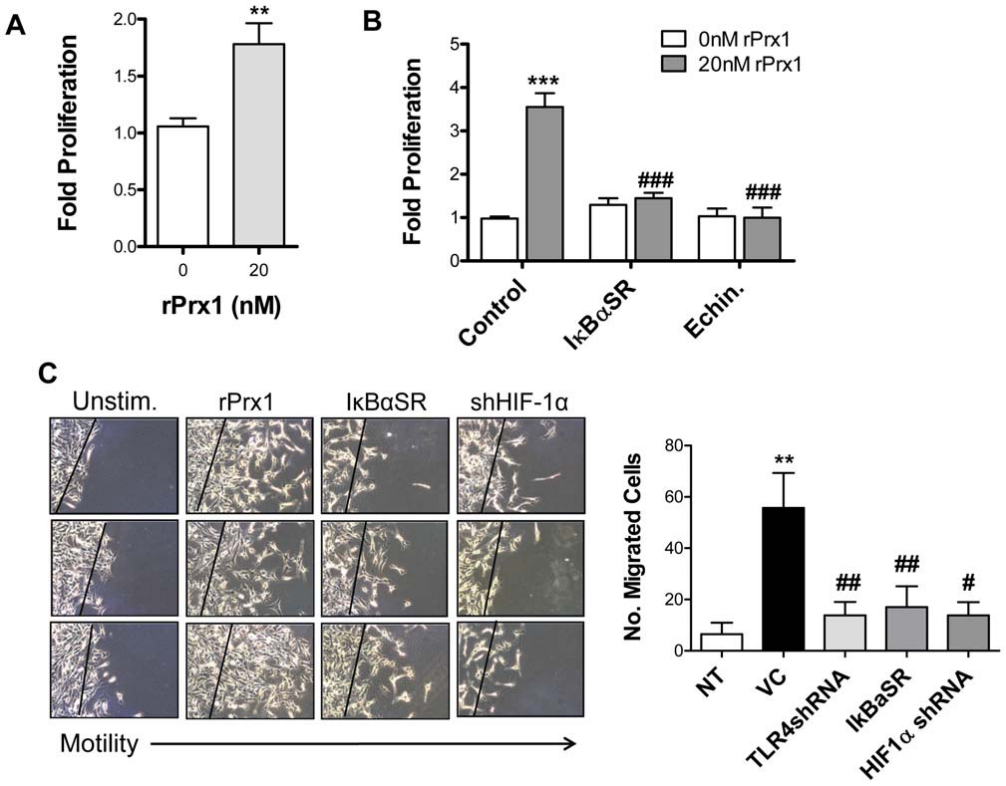
Prx1 stimulation of endothelial cell proliferation and motility depends on NF-κB, and HIF-1. **A.** HUVEC were stimulated with 20 nM Prx1 and proliferation was measured 24 hours later by SRB assay. Results are presented as the average fold proliferation over untreated control cells; error bars represent SEM. n≥3. ** Represents P≤0.001 when compared to control untreated levels. **B.** 2H-11 cells were transfected with control vectors or vectors encoding for IκB-αSR. Cells were stimulated with 20 nM Prx1 24 hours after transfection and proliferation was measured 24 hours later by MTT assay. In some cases cells were incubated with echinomycin 16 h prior to addition of Prx1. Results are presented as the average fold proliferation over untreated control cells; error bars represent SEM. n≥3. *** Represents P≤0.0001 when compared to control untreated levels; ### represents P≤0.0001 when compared to control stimulated values. **C.** 2H-11 cells were transfected with control vectors or vectors encoding for IκB-αSR or shHIF-1α. Cell migration was tested by mechanically removing cells from a section of the culture dish 24 hours after transfection and culturing the remaining cells in the absence (Unstim.) or presence of 20 nM rPrx1 for 24 hours. Images from 3 independent experiments are shown. Quantization of the number of migrated cells is shown to the right. Results are presented as the average number of cells; error bars represent SEM. n≥3. ** Represents P≤0.01 when compared to control untreated levels; ## represents P≤0.01 when compared to control stimulated values; # represents P≤0.05 when compared to control stimulated values.

Prx1 stimulates endothelial cell proliferation (HUVEC; Figure 9A), migration and differentiation of via its interaction with TLR4 [13]. To test whether these functions were dependent upon NF-κB and/or HIF-1 2H-11 endothelial cells were transfected with vectors engineered to express IκB-αSR or shHIF-1α or cells were pretreated with echinomycin prior to the addition of rPrx1. Inhibition of NF-κB or HIF-1 resulted in an abolishment Prx1 enhanced endothelial cell proliferation (Figure 9B). Similarly expression of IκB-SR or shHIF-1α inhibited Prx1 stimulation of endothelial cell migration (Figure 9C). Thus TLR4 dependent induction of endothelial cell proliferation and migration depends on NF-κB and HIF-1.

## Discussion

**Figure 10:**
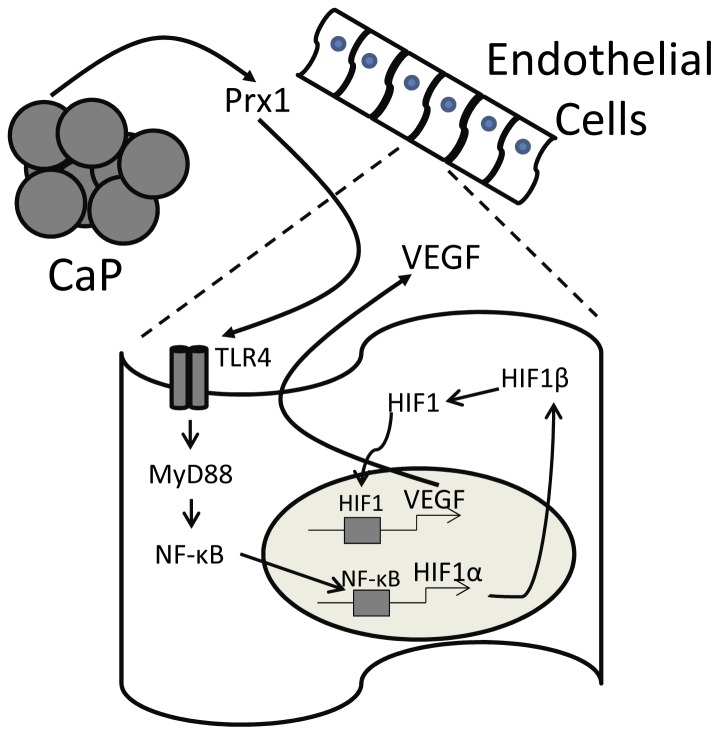
Prx1 secreted from prostate tumor cells stimulates VEGF release from endothelial cells. A schematic model is shown in which Prx1 secreted from prostate cancer cells (CaP) interact with TLR4 expressed on the surface of endothelial cells. Interaction of Prx1 with TLR4 results in activation of NF-κB and stimulation of HIF-1α gene expression. HIF-1α interacts with HIF-1β to form HIF-1. HIF-1 migrates into the nucleus and stimulates expression of VEGF, which in turn stimulates proliferation and migration of endothelial cells.

The results described above show that Prx1 enhancement of endothelial cell expression of VEGF in normoxic conditions occurs via TLR4 dependent increases in HIF-1α transcription. Induction of HIF-1α promoter activity by Prx1:TLR4 interaction was mediated by NF-κB (Figure 10). Increased HIF-1α protein stabilization appeared to be due to TLR4 activation of ERK signaling pathway. Prx1 mediated endothelial cell proliferation and migration was dependent upon NF-κB and HIF-1. Although the current studies were performed with cultured endothelial cell lines rather than CaP derived endothelial cells, the results suggest a mechanism by which Prx1 may regulate CaP angiogenesis and growth.

Previous studies examining induction of VEGF expression in macrophages by TLR4 activation have shown varying results [18]. In some studies, induction of VEGF and HIF-1α is dependent upon NF-κB activation of HIF-1α [19]–[24]; in other studies induction is dependent upon reactive oxygen [25]. Studies in fibroblasts have shown that TLR4 induction of VEGF can occur independently of HIF-1 [27]. The current study clearly demonstrates that Prx1 induction of VEGF expression by endothelial cells is dependent upon NF-κB induction of HIF-1α; inhibition of NF-κB activity by expression of a super repressor of IκB abrogated Prx1 induction of HIF-1 activity and VEGF. Thus it appears that like macrophages [13], VEGF expression by endothelial cells following TLR4 activation is dependent upon NF-κB activation of HIF-1.

VEGF expression in macrophages following exposure to LPS is synergistically enhanced by agonists of the adenosine A_2A_ receptor [22], [42]. Although our studies demonstrate a requirement for TLR4 in Prx1 induction of VEGF expression by endothelial cells, we cannot rule out involvement of additional receptors. Our previous studies have shown that Prx1 interaction with TLR4 is optimal in the presence of MD2 and CD14 [15]. MD2 is a soluble protein that associates with the extracellular portion of TLR4 and is critical for ligand binding [43]–[45]. CD14 is a co-receptor for TLRs1–4 and TLR6. CD14 has recently been shown to signal independently of TLRs and activate Src family kinases leading to activation of PLCγ2, which can trigger NF-κB activation [46]. The importance of TLR4 independent CD14 signaling in the induction of VEGF by Prx1 is unclear. Furthermore, our previous study indicated that Prx1 may bind additional receptors that have not been identified [15]. These receptors could synergize with TLR4 to promote VEGF expression in endothelial cells. It is important to note that numerous studies examining TLR4 induction of VEGF expression in macrophages and dendritic cells did not indicate a role for additional receptors [19]–[24].

Prx1 is secreted from CaP cells [13], [15]. We have shown that reduction of Prx1 expression by CaP cells correlated with a reduction in VEGF expression and formation of functional tumor vasculature [13]. The tumors used in the previous study were subcutaneous; VEGF and tumor vasculature was assessed in tumors that were not necrotic and did not contain hypoxic areas [13]. The current study shows that Prx1 induces VEGF in normoxic settings via activation of HIF-1α and that tumors with reduced Prx1 have lower levels of HIF-1α.

The ability of a tumor derived ligand for TLR4 such as Prx1 to drive VEGF expression in normoxic conditions has implications for our understanding of the role inflammation plays in tumor development and progression. VEGF expression is correlated with poor patient prognosis in many cancers including CaP [47]–[52]. Chronic inflammation of the prostate (prostatitis) leads to development of proliferative inflammatory atrophy (PIA) lesions that can transition to prostatic intraepithelial neoplasia (PIN) [53], [54]. Both types of pre-malignant lesions have potential to progress to invasive carcinoma. The transition from PIA/PIN to CaP is dependent upon an angiogenic switch [55]; VEGF is a critical regulator of the angiogenic switch and facilitates the transition from PIN to CaP [56]. The degree of hypoxia present in prostatitis is uncertain [57]; however the current study suggests that activation of the TLR4 signaling pathway can lead to expression of VEGF, regardless of oxygen status, potentially augmenting disease progression. Interestingly in the spontaneous autochthonous transgenic adenocarcinoma of mouse prostate (TRAMP) model, Prx1 expression is increased at the time of transformation [13], while HIF-1α and VEGF expression is elevated in PIN lesions [7]. Thus it is possible that secretion of Prx1 by transformed prostate cells contributes to induction of VEGF and promotion of CaP development prior to formation of areas of tumor hypoxia.

NF-κB can induce HIF-1α in macrophages [19]–[22] and endothelial cells, as shown here. HIF-1 can increase transcription and activity of NF-κB (this study and [36], [37]). Additionally, TLR4 is a HIF-1 target gene [58] and Prx1 gene expression is induced by TLR4 activation [59]. Therefore secretion of Prx1 by CaP cells has the potential to result in a self-perpetuating loop that promotes angiogenesis and chronic inflammation in established tumors. Inhibition of inflammation has been shown to reduce tumor incidence and in some cases, slow tumor progression [2]. We have shown that Prx1 interaction with TLR4 promotes inflammation [15] and angiogenesis [13]. It is possible that Prx1 may serve as a novel target for treatment of CaP. Disruption of Prx1 interaction with TLR4 has the potential to inhibit Prx1 augmentation of a pro-tumor microenvironment and stimulation of tumor angiogenesis. Prx1 interaction with TLR4 is dependent on its chaperone activity and/or decameric structure [15]. Targeted inhibition of Prx1 decameric structure/chaperone activity with small molecule inhibitors may reduce chronic inflammation, inhibit VEGF expression and limit angiogenesis in CaP.

In conclusion, the results reported here demonstrate that Prx1, an endogenous ligand for TLR4 that is secreted by CaP cells, induces VEGF expression by endothelial cells via NF-κB dependent activation of HIF-1α. Prx1 stimulation of NF-κB and HIF-1α results in endothelial cell proliferation and migration. These results suggest a mechanism by which Prx1 regulates angiogenesis in CaP and suggests a scenario of continued stimulation of angiogenesis and inflammation in CaP.

## Materials and Methods

### Ethics Statement

The RPCI Institutional Animal Care and Use Committee approved both animal care and experiments.

### Materials

Bovine serum albumin (BSA), cyclohexamide, and antibodies specific for β-actin were obtained from Sigma-Aldrich (St. Louis, MO). Echinomycin and Mithramycin A were purchased from Enzo Life Sciences International (Plymouth Meeting, PA). Antibodies specific for IκB-α, phosphorylated- IκB-α (Ser 32/36), eIF-4E, phosphorylated-eIF-4E (Ser 209) and the MEK1/2 inhibitor U0126 were purchased from Cell Signaling Technology (Boston, MA). Antibodies against ERK1/2, phosphorylated ERK1/2 (Thr 177), Laminin β-1 and VEGF were purchased from Santa Cruz Biotechnology (Santa Cruz, CA). The antibody specific for HIF-1α was purchased from Novus Biologicals (Littleton, CO). ELISAs specific for the detection of mouse and human VEGF were obtained from R&D Systems (Minneapolis, MN). The antibody specific for Prx1 was obtained from Lab Frontier (Seoul, South Korea) and does not recognize other Prx isoforms [15].

### Recombinant Prx1

Recombinant Prx1was purified as described previously using sequential ion exchange chromatography and size exclusion chromatography [60], [61]. Endotoxin levels of purified Prx1 were quantified with a Limulus Amebocyte Lysate Assay (Lonza, Walkersville, MD) according to manufacturer's directions. Purified Prx1 was found to contain 14.14±0.050 EU/ml. Although not depicted all experiments included controls in which rPrx1 was boiled or polymixin B, an inhibitor of LPS, was added in combination with rPrx1 [15]. Boiling of rPrx1 eliminated its effects; addition of polymixin B had no effect on rPrx1 effects.

### Animals

Severe combined immunodeficiency (SCID) pathogen-free mice were purchased through the Laboratory Animal Resource of RPCI. Animals were housed in individually ventilated microisolator cages in laminar flow units under ambient fluorescent light. The mice were maintained in a limited access barrier facility at RPCI. The RPCI Institutional Animal Care and Use Committee approved both animal care and experiments.

### Cell Lines

2H-11 and HUVEC cell lines were obtained from American Type Culture Collection (ATCC; Manassas, VA). 2H-11 were cultured in DMEM supplemented with 10% defined FBS, 100 U/ml penicillin, and 100 ug/ml streptomycin at 37°C and 10% CO_2_. HUVEC were cultured in supplemented endothelial growth media (Promega, Madison, WI) at 37°C and 5% CO_2_. Retroviral short hairpin RNA expression constructs [62] specific for Prx1 expression (shPrx1) and non-specific (scramble) were used to reduce Prx1 expression in the PC-3M cell lines [63]. Stable scramble/shPrx1 cell lines were maintained in the presence of 125 µg/mL G418 (Manassas, VA).

2H-11 cells were transfected with vectors encoding a MyD88 dominant negative (DN) [64] using FuGENE 6 (Invitrogen, Carlsbad, CA) according to the manufacturer's protocol. Cells were similarly transfected with an IκB-α super repressor (IκB-αSR) [65], HIF-1 Luciferase reporter plasmid [66] and a NF-κB Luciferase reporter plasmid [67] all received as a generous gift from Dr. Eugene Kandel (Roswell Park Cancer Institute). Short hairpin RNA constructs specific to TLR4 and HIF-1α expression were purchased from S.A. Biosciences (Fredrick, MD) and were transfected using FuGENE6 according to the manufacturer's instructions. Transfection with shRNA constructs led to a greater than 70% reduction in target protein expression.

### VEGF Promoter Constructs

The known VEGF promoter region stretches 1217 bps upstream of the transcription initiation site. The VEGF promoter region was isolated from mouse genomic DNA by PCR using primers listed in Table 1. The resulting product was ligated into the pGL4.14 luciferase vector (Promega; Madison, WI). Deletion constructs were generated by using the 1217 bp known VEGF promoter as a template and deletion specific primers (Table 1). The products were ligated into the pGL4.14 Luciferase. Mutations of the promoter elements in the VEGF promoter were performed by site directed mutagenesis according to the manufacturer's instructions (Stratagene Cloning Systems, La Jolla, CA). Mutagenesis primers are listed in Table 1. The final VEGF promoter constructs were sequenced to confirm identity.

**Table 1:** Primer Sequences.

Primer Name	Strand	Sequence
1217	5′	5′-AGTCCCTCGAGTGTTTAGAAGATGAACCGTAAGCCT-3′
1217	3′	5′-CTCAAGCTTTCTTTCTCACCGGTAACAGTAACAGCGGTGG-3′
1000	5′	5′-CATCTCGAGGTGTGTGTGTGTGTGTGAGAG-3′
850	5′	5′-CCCCTCGTGCAGTGCCACAAATTTGGTGCC-3′
504	5′	5′-GGTCTCGAGAATGTAGTCACTAGGGGGCGC-3′
400	5′	5′-TGCCTCGAGGCTGTGTGTGTGTGTGTAGTG-3′
85	5′	5′-GGCCTCGAGAAAGGCGGTGCCTGGCTCCAC-3′
S1	5′	5′-CCACCAGACCGTCCCCGGAAGGTCTGGGCGGGGC-3′
S2	5′	5′-GGGGCGGGTCTGGGAAGGGCTTGGGGGTGGAG-3′
S3	5′	5′-GGCGGGGCTTGGGGAAGGAGCTAGATTTCCTC-3′
N1	5′	5′-CCCCTGATTCCCAATACTCTGGGAATTAACAGTGTGTCCTGAGC-3′
N2	5′	5′-CCCCTGATTCCCAATACTCTGAAATTCCCAGTGTGTTCCTGAGC-3′
N3	5′	5′-CGCGGTAGTGGCCTAGAAGCTCCCCGAAAGGCTGCC-3′
N4	5′	5′-CGGTAGTGGCCTAGGGGCTCTTCGAAAGGCGGTGCC-3′
HRE^MUT^	5′	5′-CCAGACTACAAGTGCATACACGGGTTTCCACAGGTCG-3′

### HIF-1α Promoter Constructs

The known HIF-1α promoter region stretches 800 bps upstream of the transcription initiation site. The HIF-1α promoter region was constructed by Genescript (Piscataway, NJ) and cloned into a pGL4.14 luciferase reporter vector and sequenced to confirm its identity.

### Luciferase Assays

All plasmids were prepared using an Invitrogene Midiprep Kit (Qbiogene, Carlsbad, CA). 2H-11 cells were plated in 24 well plates at a density of 1×10^5^ cells/well and transiently transfected using FuGENE as described previously. Firefly luciferase reporter systems included the VEGF promoter, the HIF-1 responsive promoter, the HIF-1α promoter region and the NF-κB responsive promoter. Cells were cotransfected with the *Renilla* luciferase vector, phRL-CMV (Promega) in order to normalize for transfection efficiency. Similarly, when indicated cells were also transfected with vectors encoding shTLR4, MyD88DN, shHIF-1α, and IκB-αSR. After 24 hour incubation, the transfectants were suspended in fresh medium and stimulated with rPrx1 (20 nM). In some experiments cells were incubated with echinomycin (10 nM) for 16 h or mithramycin A (1 nM) for 24 h prior to addition of Prx1. Cells were lysed with passive lysis buffer (Promega) 6 h after Prx1 addition and luciferase assays were performed using the Dual luciferase Assay Kit (Promega) following the manufacturer's protocol. Luciferase light units were measured using L_max_ Luminescence injection port Reader (Molecular Devices, Sunnyvale, CA) using dual injector system. Firefly luciferase light units were normalized to *Renilla* luciferase light units. The results are reported as relative activity (Firefly luciferase activity/*Renilla* luciferase activity) and are representative of at least three independent experiments.

### Quantitative PCR

Total RNA was extracted from 2H-11 cells using Trizol ® reagent (Invitrogen Corporation, USA) according to the manufacturer's directions. The RNA preparations were quantified by A_260_ measurement on a spectrophotometer (BioPhotometer, Eppendorf, Germany), and stored at −20°C. The quality of RNA samples was determined by agarose gel electrophoresis through and staining with ethidium bromide. 18S and 28S RNA bands were visualized under a UV light. The OD_260_/OD_280_ ratios of the samples were shown to be between 1.8 and 2.0.

Real-time quantitative PCR was performed using the relative standard curve method to analyze the target gene expression. Isolated cellular RNA was used as the template for the two step real-time quantitative PCR which was performed in the Applied Biosystem 7500 Real-Time PCR System (Applied Biosystems, Foster City, CA) in a 25 µl reaction volume using the one-step SYBR® Green qPCR kit (Invitrogen, Carlsbad, CA), according to the manufactures recommendations. The primer sequences for VEGF, HIF-1α, HIF-1α ChIP assays and GAPDH were taken from previous publications [20], [26], [49]. For the VEGF ChIP assay, the HIF-1 mutated 3′ primer and the VEGF promoter 5′ primers were used. Real-time quantitative PCR was performed at 94°C for 5 min, followed by 40 cycles at 95°C for 30 s, 65°C for 40 s, and 72°C for 40 s. Fluorescence was measured following each cycle and analyzed by ABI PRISM 7000 sequence detection system version 1.3.1 (Applied Biosystems, Foster City, CA). Specificity of PCR products was confirmed by melting curve analyses and agarose gel analyses. For each sample analyzed, the mean 2^−Δ*C*t^ value based on the results of all experiments was calculated, together with that of the corresponding standard samples. Relative amount of gene mRNAs were normalized for loading differences by GAPDH gene mRNA. All samples were treated in duplicate and experiments were repeated in triplicate. Results are reported as relative VEGF mRNA expression.

### Immunohistochemistry

PC-3M scramble/shPrx1 tumors (150 mm^3^) were harvested, fixed in zinc-formalin (R&D Systems), and paraffin embedded. HIF-1α and Prx1 expression in PC-3M scramble/shPrx1 tumors was performed by the immunohistochemistry facility at Roswell Park Cancer Institute as previously described [68]. Slides were scanned with the ScanScope XT System (Aperio Technologies Inc, Vista CA). Scans were then digitally viewed using ImageScope Analysis software.

### Endothelial Cell Proliferation, Migration, and Differentiation

Endothelial cell proliferation assays were performed as previously described [69]. In brief, 10^3^ 2H-11 endothelial cells were plated per well in a 96-well dish. In indicated experiments cells were transfected using FuGENE 6 (Invitrogen, Carlsbad, CA) with vectors engineered to express a MyD88DN or a super IκB-α repressor of NF-κB. Recombinant Prx1 (0, 20, 50 nM) was used to stimulate the endothelial cells for 24 hours. Cell proliferation was measure by either 3-(4,5-Dimethylthiazol-2-yl)-2,5-diphenyltetrazolium bromide (MTT) [70] or sulforhodamine B (SRB) [71] colorimetric assay.

Endothelial motility assays were performed according to Takata et al [72]. In brief, 2.5×10^5^ 2H-11 endothelial cells were plated at in a 10 cm dish. Endothelial cells were transfected according to the manufactures instructions for FuGENE 6 (Invitrogen, Carlsbad, CA) with vectors engineered to express shRNA specific to TLR4, an IκB-αsuper repressor of NF-κB and shRNA specific to HIF-1α. Two voids of approximately 2.0 cm in width were created by scraping plates with cell spatulas. Cultured cell media was replaced and in the indicated experiments, cells were treated with rPrx1 (0, 20, 50 nM). The effect of Prx1 on endothelial cell motility was analyzed 24 hours later on ten random digital images (10× magnification) from each plate. Experiments were performed in duplicate and in three separate trials.

### Molecular Analyses

ELISA kits specific for mouse and human VEGF were purchased from R&D systems (Minneapolis, MN) and assays were completed according to manufacturer's instructions. The mouse/human Prx1 ELISA kit was purchased from Northwest Life Science Specialties, LLC (Vancouver, WA) and assays were completed according to manufacturer's instructions. Lysates generated by mammalian lysis buffer (SIGMA), were separated by electrophoresis and immunoblotted with antibodies specific for VEGF, HIF-1α, NF-κB, phosphorylated IκB-α, IκB-α, phosphorylated STAT3, STAT3, phosphorylated ERK1/2, ERK1/2, phosphorylated eIF-4E and eIF-4E expression. Proteins were detected with the ECL system (BioRad).

Cytoplasmic and nuclear lysates were prepared as described previously [63]. Briefly, cell pellets were suspended in a cytoplasmic, low salt buffer [1% Triton ×100, 10 mM HEPES (pH 7.9), 10 mM HEPES (PH 7.9), 10 mM KCl, 1 mM EDTA, 1 mM EGTA, 1 mM DTT, 1 mM PMSF, 1 µg/mL] and incubated for 10 min incubation on ice. Samples were spun at 6000 rpm for 30 minutes; the resulting supernatant represented the cytoplasmic fraction. Nuclei were lysed in high salt buffer [10% glycerol, 50 mM HEPES, pH 7.9, 400 mM KCl, 1 mM EDTA, 1 mM EGTA, 1 mM DTT, mM PMSF, 1 µg/mL each of aprotinin and leupeptin, and 1 mM Na3VO4] by vigorous vortexing for 20 min on ice. Nuclear extract was collected by centrifugation at 12,000 rpm for 15 min. All the centrifugation steps were performed at 4°C.

### Statistical Analysis

Statistical analyses were performed using a standardized Student's t-test with Welch's correction, where equal variances were not assumed, to compare experimental groups. Differences were considered significant when P values were ≤0.05.
